# Serotonin modulation of cortical neurons and networks

**DOI:** 10.3389/fnint.2013.00025

**Published:** 2013-04-19

**Authors:** Pau Celada, M. Victoria Puig, Francesc Artigas

**Affiliations:** ^1^Department of Neurochemistry and Neuropharmacology, Institut d' Investigacions Biomèdiques de Barcelona (CSIC), IDIBAPSBarcelona, Spain; ^2^Centro de Investigación Biomédica en Red de Salud Mental (CIBERSAM)Madrid, Spain; ^3^The Picower Institute for Learning and Memory, Massachusetts Institute of TechnologyCambridge, MA, USA

**Keywords:** 5-hydroxytryptamine or serotonin, dorsal raphe nucleus, electrophysiological recordings, GABAergic interneurons, oscillatory activity, prefrontal cortex, pyramidal neurons, serotonin receptors

## Abstract

The serotonergic pathways originating in the dorsal and median raphe nuclei (DR and MnR, respectively) are critically involved in cortical function. Serotonin (5-HT), acting on postsynaptic and presynaptic receptors, is involved in cognition, mood, impulse control and motor functions by (1) modulating the activity of different neuronal types, and (2) varying the release of other neurotransmitters, such as glutamate, GABA, acetylcholine and dopamine. Also, 5-HT seems to play an important role in cortical development. Of all cortical regions, the frontal lobe is the area most enriched in serotonergic axons and 5-HT receptors. 5-HT and selective receptor agonists modulate the excitability of cortical neurons and their discharge rate through the activation of several receptor subtypes, of which the 5-HT_1A_, 5-HT_1B_, 5-HT_2A_, and 5-HT_3_ subtypes play a major role. Little is known, however, on the role of other excitatory receptors moderately expressed in cortical areas, such as 5-HT_2C_, 5-HT_4_, 5-HT_6_, and 5-HT_7_. *In vitro* and *in vivo* studies suggest that 5-HT_1A_ and 5-HT_2A_ receptors are key players and exert opposite effects on the activity of pyramidal neurons in the medial prefrontal cortex (mPFC). The activation of 5-HT_1A_ receptors in mPFC hyperpolarizes pyramidal neurons whereas that of 5-HT_2A_ receptors results in neuronal depolarization, reduction of the afterhyperpolarization and increase of excitatory postsynaptic currents (EPSCs) and of discharge rate. 5-HT can also stimulate excitatory (5-HT_2A_ and 5-HT_3_) and inhibitory (5-HT_1A_) receptors in GABA interneurons to modulate synaptic GABA inputs onto pyramidal neurons. Likewise, the pharmacological manipulation of various 5-HT receptors alters oscillatory activity in PFC, suggesting that 5-HT is also involved in the control of cortical network activity. A better understanding of the actions of 5-HT in PFC may help to develop treatments for mood and cognitive disorders associated with an abnormal function of the frontal lobe.

## Introduction

Serotonin (5-hydroxytryptamine, 5-HT) is one of the phylogenetically older molecules used in cellular communications. It is present in the central nervous system (CNS) of vertebrates and invertebrates and plays the role of neurotransmitter/neuromodulator. It also functions as a developmental signal in the CNS and regulates a variety of physiological functions in the periphery (where most 5-HT is present), such as intestinal motility, platelet aggregation, and vasoconstriction.

Within the CNS, the serotonergic system is involved in a large number of functions resulting from its widespread innervation of the whole neuraxis. The axons of serotonergic neurons of the midbrain raphe nuclei reach almost every brain structure. Action potentials traveling along these axons release 5-HT which can act on pre- and postsynaptic receptors, coupled to different signal transduction mechanisms. So far, 14 different 5-HT receptor subtypes have been identified, corresponding to 7 different families: 5-HT_1_ (5-HT_1A_, 5-HT_1B_, 5-HT_1D_, 5-HT_1E_, 5-HT_1F_), 5-HT_2_ (5-HT_2A_, 5-HT_2B_, 5-HT_2C_), 5-HT_3_, 5-HT_4_, 5-HT_5_ (5-HT_5A_, 5-HT_5B_), 5-HT_6_, and 5-HT_7_. With the exception of the 5-HT_3_ receptor, a pentameric ligand-gated ion channel composed of several subunits (up to 5 different ones have been identified), the rest of 5-HT receptors belong to the superfamily of G-protein coupled receptors and their activation results mainly in modulatory actions in the neurons expressing these receptors.

Given the widespread innervation of the brain and the richness of signals evoked by 5-HT, it is not surprising that the 5-HT system is the target of many drugs used to treat brain diseases and also of recreational drugs. For instance, most antidepressant treatments block the 5-HT transporter and increase the extracellular (or synaptic) 5-HT concentration and hence, they indirectly elevate the serotonergic tone at pre- and postsynaptic 5-HT receptors. This action is supposed to mediate the therapeutic effect of these drugs. Moreover, some anxiolytic drugs are 5-HT_1A_ receptor agonists and 5-HT_3_ receptor antagonists are commonly used to treat emesis induced by anti-cancer treatments.

On the other hand, drugs of abuse such as cocaine, amphetamine or MDMA (ecstasy) target monoaminergic transporters, including the 5-HT transporter. Furthermore hallucinogens like LSD, DOI, DOB, or DOM are 5-HT_2_ receptor agonists whereas atypical antipsychotics act as preferential antagonists of these receptors [(Roth et al., 2004); see Geyer and Vollenweider (2008) for a review].

Among the various 5-HT receptors, the 5-HT_1_ family has probably received the largest attention because of the high density expression in limbic (5-HT_1A_) and motor (5-HT_1B_) brain areas and the various roles subserved by some of its members. Thus, in addition to being located postsynaptically to 5-HT axons, 5-HT_1A_ and 5-HT_1B_ receptors are autoreceptors in 5-HT neurons and therefore control the overall (5-HT_1A_) as well as the local (5-HT_1B_) activity of the system. 5-HT_1B_ receptors are also terminal heteroreceptors and modulate the release of various transmitters, including dopamine, glutamate, GABA, and acetylcholine. Moreover, 5-HT_1A_ receptors are highly expressed by different neuronal types (mainly pyramidal but also GABAergic) in prefrontal cortex (PFC), which suggests an important role in the control of mood and emotions as well as in cognitive processes. An extensive review of the characteristics of the serotonergic system is beyond the scope of the present review. The reader is referred to several review papers dealing with the anatomy, physiology, neurochemistry, and neuropharmacology of the 5-HT system (Jacobs and Azmitia, 1992; Barnes and Sharp, 1999; Adell et al., 2002; Smythies, 2005; Artigas, 2013). In the following sections, we focus on the role of 5-HT in the modulation of cortical activity.

## The cortical 5-HT system: receptor localization

There is growing evidence that the serotonergic pathways originating in the dorsal and median paphe nuclei (DR and MnR, respectively) are critically involved in cortical functions. 5-HT appears to play an important role in the development of the somatosensory cortex and formation of the barrel cortex. In adult brain, the axons of 5-HT neurons innervate a large number of cortical areas, including the entorhinal and cingulate cortices, which contain a moderate to high density of 5-HT receptors. However, of all cortical regions, the frontal lobe is the richest area in serotonergic terminals and 5-HT receptors.

Yet, unlike dopamine, whose function in the PFC has been extensively studied (Williams and Goldman-Rakic, 1995; Robbins and Arnsten, 2009), the role of 5-HT in PFC remains less known than that of dopamine. Indeed, the widespread localization of 5-HT receptors (particularly of the 5-HT_1A_, 5-HT_2A_, and 5-HT_2C_ subtypes) and the high density of 5 HT axons (greater than in any other cortical area) in this cortical region suggest an important role of 5-HT in cognitive and emotional functions depending on PFC activity. Hence, the selective depletion of 5-HT in the monkey frontal cortex impairs cognitive flexibility (increases perseveration), and reversal learning (Clarke et al., 2004, 2005), likely via 5-HT_2A_ receptors (Carli et al., 2006; Boulougouris et al., 2007). In addition, optimized levels of 5-HT in the PFC are important for behavioral inhibition, as elevated or reduced 5-HT increases impulsivity (Harrison et al., 1997; Dalley et al., 2002; Winstanley et al., 2004). In fact, both stimulation of 5-HT_1A_ receptors and blockade of 5-HT_2A_ receptors decrease impulsivity (Winstanley et al., 2003; Carli et al., 2006; Talpos et al., 2006), suggesting that a downregulation of cortical serotonergic activity may effectively promote behavioral control. 5-HT in the frontal cortex is also involved in the modulation of attention in humans, an effect that implicates 5-HT_1A_, but not 5-HT_2A_, receptors (Carter et al., 2005; Scholes et al., 2006). Moreover, as observed for dopamine D1 receptors (Williams and Goldman-Rakic, 1995; Puig and Miller, 2012), the blockade of 5-HT_2A_ receptors in the monkey lateral PFC avoids the increase in neuronal activity during a working memory task (Williams et al., 2002), and a study associates allelic variants of this receptor with memory capacity in humans (De Quervain et al., 2003). Furthermore, hallucinogens like LSD or DOI are 5-HT_2A_ receptor agonists, which also suggests a role of 5-HT in the processing of external (sensory) and internal information through the activation of 5-HT_2A_ receptors. On the other hand, 5-HT_1A_ agonists display anxiolytic/antidepressant activity in animal models (Martin et al., 1990; De Vry, 1995; Carr and Lucki, 2011) whereas 5-HT_1A_ receptor antagonists reverse drug-induced cognitive deficits (Harder and Ridley, 2000; Mello e Souza et al., 2001; Misane and Ögren, 2003). Likewise, preclinical studies suggest that 5-HT_4_ receptor agonists may exert rapid antidepressant actions by acting on PFC receptors (Lucas et al., 2007).

One of the key basic information relevant for the interpretation of physiological and behavioral data concerning the cortical 5-HT system is the regional and cellular localization of the 5-HT receptors. Several studies have examined the localization of 5-HT in the cortex. Early studies using receptor autoradiography and *in situ* hybridization enabled to identify the presence of various 5-HT receptors in cortical areas, notably the 5-HT_1A_, 5-HT_2A_, and 5-HT_2C_ subtypes (Pazos and Palacios, 1985; Pazos et al., 1985; Pompeiano et al., 1992, 1994). Further studies identified the presence of other receptor subtypes, yet in lower density than these ones.

5-HT_1A_ receptors are particularly enriched in the rodent medial PFC (mPFC), entorhinal cortex and, to a lesser extent, cingulate and retrosplenial cortices. Outside the cortex, they are densely expressed in the hippocampus, septum and the raphe nuclei. In the latter location, the receptor is almost exclusively expressed by 5-HT neurons, where it functions as an autoreceptor in the plasma membrane of perikarya and dendrites (Riad et al., 2000). PET scan studies using a radiolabeled selective antagonist ([^11^C]-WAY-100635) have shown a very similar distribution in human brain, with an enrichment of the signal in the temporal and frontal lobes, cingulate cortex and the raphe nuclei (Martinez et al., 2001). Interestingly, as also observed in rats (Weber and Andrade, 2010), there is a marked rostro-caudal negative gradient in the abundance cortical of 5-HT_1A_ receptors, with the largest abundance in PFC.

Likewise, the neocortex of rodent, primate and human brains show a large abundance of 5-HT_2A_ receptors, with an enrichment in frontal regions (Pompeiano et al., 1994; Burnet et al., 1995; López-Giménez et al., 1998; Hall et al., 2000; Amargós-Bosch et al., 2004). Lower abundances are found in ventro-caudal part of CA3, medial mammillary nucleus, striatum (dorsal and ventral) and several brainstem nuclei (Pompeiano et al., 1994; Burnet et al., 1995; López-Giménez et al., 1998). Interestingly, pyramidal neurons in the rat PFC that simultaneously project to the ventral tegmental area and the dorsal raphe nucleus express 5-HT_2A_ receptors (Vázquez-Borsetti et al., 2009, 2011). This reveals a close anatomical interaction or “loop” between frontal areas and dopamine and serotonin neurons of the brainstem, as found in several electrophysiological studies (Thierry et al., 1979, 1983; Tong et al., 1996; Hajós et al., 1998; Celada et al., 2001; Martín-Ruiz et al., 2001). As for 5-HT_1A_ receptors, there is a good agreement between the autoradiographic and *in situ* hybridization signals, which indicates that the receptor is expressed mainly in the somatodendritic region. Similar regional distributions have been reported in human brain using the selective antagonist ligand M100907 *in vivo* (PET scan) or *in vitro* (autoradiography) (Hall et al., 2000).

5-HT_1A_ and 5-HT_2A_ receptors are present in a high proportion of cells in some cortical regions. Double *in situ* hybridization studies, to label the cellular phenotype and the respective receptor mRNA, have shown that around 50% of pyramidal neurons (labeled with the vGluT1 mRNA) and 20–30% of GABAergic interneurons (labeled with GAD65/67 mRNA) express 5-HT_1A_ and/or 5-HT_2A_ receptor mRNAs in various areas of the PFC (Santana et al., 2004) (Table 1). Interestingly, about 30% of parvalbumin-expressing fast-spiking interneurons in the PFC express 5-HT_1A_ or 5-HT_2A_ receptors which, unlike pyramidal neurons, are largely distributed in separate neuron populations (Puig et al., 2010).

**Table 1:** **Proportion of pyramidal and local GABAergic neurons that express the mRNAs encoding 5-HT_1A_ and 5-HT_2A_ receptors**.

	**Pyramidal neurons**		**GABAergic neurons**	
	**5-HT**_**1A**_	**5-HT**_**2A**_	**5-HT**_**1A**_	**5-HT**_**2A**_
	**mRNA**	**mRNA**	**mRNA**	**mRNA**
MOs	54 ± 4	60 ± 2	28 ± 6	28 ± 10
ACAd	54 ± 3	66 ± 5	22 ± 4	32 ± 2
PrL	61 ± 2	51 ± 3	20 ± 1	34 ± 1
ILA^a^	40 ± 4^*^	12 ± 1^**^	22 ± 4	22 ± 3
TT	63 ± 6	81 ± 3^***^	24 ± 1	24 ± 2
PIR	60 ± 2	50 ± 3	21 ± 6	24 ± 2
Layer VI	54 ± 3	26 ± 3^+^	23 ± 4	11 ± 3^++^

Data are means of three rats and represent the percentage of the counted cells expressing the mRNAs of each 5-HT receptor in pyramidal (vGluT1 mRNA-positive) and GABAergic (GAD mRNA-positive) cells. MOs, secondary motor area; ACAd, dorsal anterior cingulate area; PrL, prelimbic area; ILA, infralímbic area; PIR, piriform cortex; TT, tenia tecta. Layer VIa denotes deep areas of the sensorimotor cortex at prefrontal level.

a *The data of the ILA correspond to its more ventral part, which shows a remarkable low level of 5-HT*_2A_ *receptor, whereas cell counts from its dorsal part are more similar to those of PrL*.

* P < 0.05 vs. PrL, TT, and PIR;

** *P < 0.05 vs. the rest of areas, except layer VIa (P = 0.9)*;

*** *P < 0.05 vs. the rest of areas*;

+ *P < 0.05 vs. the rest of areas except ILA*;

++ *P < 0.05 vs. ACAd and PrL (Tukey test post-ANOVA). Data from Santana et al. (2004)*.

**Figure 1:**
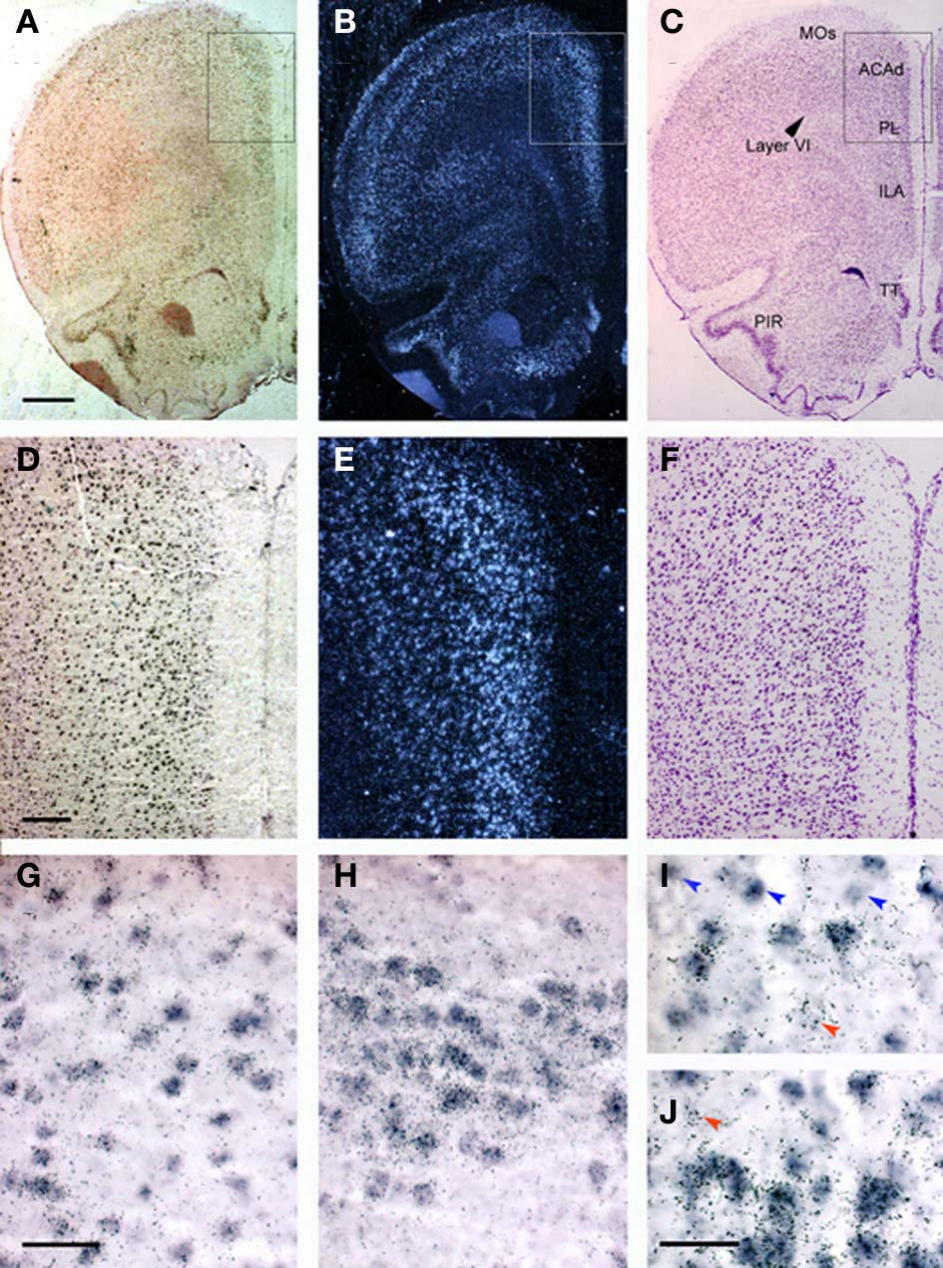
**Localization of 5-HT_1A_ and 5-HT_2A_ receptor mRNAs in the rat PFC using double *in situ* hybridization histochemistry. (A–C)** Coronal sections of rat PFC showing a large number of cells expressing **(A)** 5-HT_1A_ receptors (Dig-labeled oligonucleotides) or **(B)** 5-HT_2A_ receptors (dark field; 33P-labeled oligonucleotides); **(C)** an adjacent Nissl-stained section. Note the abundant presence of cells expressing both receptors in layers II–V, as well as in piriform cortex (PIR) and tenia tecta (TT). **(D–F)** Enlargements of the marked area in panels **(A–C)**. **(D,E)** Show the presence of a large number of cells containing 5-HT_1A_ and 5-HT_2A_ receptor transcripts in cingulate (ACAd) and prelimbic (PL) cortex. Cells in deep layers (VI) express preferentially 5-HT_1A_ receptor mRNA. **(G–J)** Individual cells expressing both receptor transcripts. Occasional cell profiles containing only 5-HT_1A_ (blue arrowheads) or 5-HT_2A_ receptor mRNAs (red arrowheads) were seen in the infralimbic cortex **(G,I)**, piriform cortex **(H)** and taenia tecta **(J)**. Bar size is 1 mm in **(A–C)**, 250 μm in **(D–F)**, 50 μm in **(G,H)** and 30 μm in **(I,J)**. Reproduced with permission from Amargós-Bosch et al. (2004).

Figure 1 shows the localization of the transcripts for 5-HT_1A_ and 5-HT_2A_ receptors in the PFC of the rat. Interestingly, 5-HT_1A_ and 5-HT_2A_ receptor transcripts are heavily co-expressed in rat and mouse PFC. Approximately 80% of the cells expressing 5-HT_1A_ receptor mRNA also express the 5-HT_2A_ receptor mRNA in all PFC areas examined, except in layer VI and the lower part of the infralimbic area, where the density of cells expressing 5-HT_2A_ receptors is much lower (Amargós-Bosch et al., 2004; Santana et al., 2004).

The abundant co-expression of 5-HT_1A_ and 5-HT_2A_ receptors raises questions about the physiological role of the simultaneous occurrence of inhibitory (5-HT_1A_) and excitatory (5-HT_2A_) receptors responding to 5-HT in the same cortical neurons. Various hypotheses have been examined (Amargós-Bosch et al., 2004; Puig et al., 2005), but perhaps one of the most convincing explanations is the putative localization of both receptors in different cellular compartments. Thus, immunohistochemical studies by several groups—using different antibodies—consistently show a predominant location of 5-HT_2A_ receptors in the apical dendrites (and to a lower extent, cell bodies) of cortical pyramidal neurons (Jakab and Goldman-Rakic, 1998, 2000; Jansson et al., 2001; Martín-Ruiz et al., 2001), where they may amplify the impact of excitatory synaptic currents. On the other hand, there is a considerable disagreement in regards to the location of cortical 5-HT_1A_ receptors, due to the use of different antibodies. A homogenous labeling of cell bodies and dendrites was initially reported (Kia et al., 1996), but more recent studies performed in rodent, primate, and human brain tissues using a different antibody (Azmitia et al., 1996) show the *exclusive* labeling of the axon hillock of pyramidal neurons (De Felipe et al., 2001; Czyrak et al., 2003; Cruz et al., 2004). This location suggests that 5-HT axons would be able to establish axo-axonic contacts with pyramidal neurons, similar to those established by chandelier GABA interneurons, which would markedly impact on the generation of action potentials. In this way, 5-HT axons reaching apical dendrites would be able to modulate glutamatergic inputs onto pyramidal cells (Aghajanian and Marek, 1997; Puig et al., 2003) whereas those reaching axon hillocks would control the probability of generation of nerve impulses through the activation of 5-HT_1A_ receptors. However, existing discrepancies on the cellular localization of 5-HT_1A_ receptors prevent to draw firm conclusions on this point.

5-HT_1B_ and 5-HT_1D_ receptors show a widespread brain distribution, with a relative low abundance in cortex. Radioligand binding and autoradiographic studies have detected the presence of a high density of 5-HT_1B_ receptors in the basal ganglia and hippocampal formation, particularly the subiculum (Pazos and Palacios, 1985; Offord et al., 1988). They are negatively coupled to adenylate cyclase and activation of 5-HT_1B_ by selective agonists decreases the forskolin-stimulated adenylate cyclase levels [for review see Sari (2004)].

The comparison of autoradiographic, *in situ* hybridization and immunohistochemcial studies has revealed that 5-HT_1B_ receptors are located both presynaptically (i.e., on 5-HT axons) and postsynaptically to 5-HT neurons, mostly on axons of intrinsic neurons of the basal ganglia (Riad et al., 2000). Presynaptic 5-HT_1B_ autoreceptors, however, represent a small proportion of the entire population of 5-HT_1B_ receptors in the brain because the lesion of 5-HT neurons does not generally result in a reduction of their density (Compan et al., 1998). Notwithstanding the low density of cortical 5-HT_1B_ receptors seen by autoradiography, electrophysiological studies have identified 5-HT_1B_ receptor-mediated actions in the cingulate cortex of the rat (Tanaka and North, 1993; see below). The other two members of the 5-HT_1_ family (5-HT_1E_ and 5-HT_1F_ receptors) are also present in cortex, particularly entorhinal cortex, yet their low abundance and the lack of selective pharmacological tools have hampered the study of their actions on cortical neurons.

5-HT_2B_ receptors are expressed in a very low density in the brain. In contrast, 5-HT_2C_ receptors (formerly named 5-HT_1C_ receptors) are highly expressed in the choroid plexus (where they were initially identified), various cortical areas in the rodent brain, particularly the PFC, the limbic system (nucleus accumbens, hippocampus, amygdala) and the basal ganglia (caudate nucleus, substantia nigra). 5-HT_2C_ receptors are also expressed in the human cortex, yet their abundance relative to other brain areas appears to be lower than in rat brain (Clemett et al., 2000; Pandey et al., 2006). Interestingly, immunohistochemical studies suggest that cortical 5-HT_2C_ receptors are mainly expressed in pyramidal neurons (Clemett et al., 2000; Puig et al., 2010), and not in fast-spiking interneurons (Puig et al., 2010), yet data using a different antibody indicate that more than 50% of the 5-HT_2C_ receptor immunoreactivity is present in GABAergic neurons (Liu et al., 2007).

**Figure 2:**
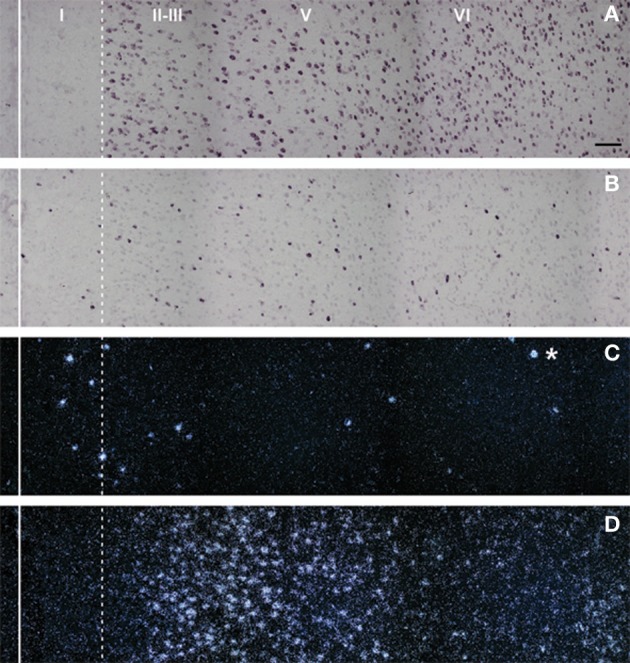
**Composite photomicrographs showing the localization of cells expressing vGluT1 (A), GAD (B), 5-HT_3_ (C), and 5-HT_2A_ (D) mRNAs through layers I–VI at the level of the prelimbic area in the rat PFC.** The continuous vertical line denotes the location of the midline whereas the dotted line shows the approximate border between layer I and II. Pyramidal neurons (as visualized by vGluT1 mRNA) are present in layers II–VI whereas GAD mRNA-positive cells are present in all layers, including layer I. Note the segregation of cells expressing 5-HT_3_ **(C)** and 5-HT_2A_ receptors **(D)**. 5-HT_3_ receptor transcript is expressed by a limited number of cells present in layers I–III, particularly in the border between layers I and II. However, they represent 40% of GABAergic neurons in layer I. On the other hand, cells in these locations, particularly in layer I, do not express 5-HT_2A_ receptors. The asterisk denotes an artifact of the emulsion, seen in the dark field. Scale bar = 150 μm. Reproduced with permission from Puig et al. (2004).

5-HT_3_ receptors are moderately abundant in the neocortex and other telecephalic regions, such as the olfactory cortex, the hippocampus, and the amygdala. Interestingly, most cortical 5-HT_3_ receptor mRNA is located in GABAergic interneurons, as assessed by *in situ* hybridization (Morales and Bloom, 1997; Puig et al., 2004). These are calbindin- and calretinin-(but not parvalbumin-) containing neurons and are located in superficial cortical layers (I–III) (Morales and Bloom, 1997; Puig et al., 2004) (Figure 2).

5-HT_4_ receptors are abundant in the olfactory tubercle, some structures of the basal ganglia (caudate putamen, ventral striatum), medial habenula, hippocampal formation, and amygdala. The neocortex contains low levels of the 5-HT_4_ receptor and its encoding mRNA, as assessed by autoradiography and *in situ* hybridization, respectively (Waeber et al., 1994; Vilaró et al., 1996, 2005). However, studies using single-cell RT-PCR technique reported that ~60% of pyramidal neurons recorded in the PFC contain the 5-HT_4_ receptor transcript (Feng et al., 2001), and overexpression of 5-HT_4_ in the mPFC increases DRN 5-HT activity (Lucas et al., 2005) likely through descending excitatory axons reaching the DR (Celada et al., 2001; Vázquez-Borsetti et al., 2011).

5-HT_5_ receptors are the less well understood 5-HT receptors. Both 5-HT_5A_ and 5-HT_5B_ receptor subtypes were found in rodents whereas only the 5-HT_5A_ was identified in human brain. The 5-HT_5A_ receptor mRNA is found in relative high levels in the hippocampus, the medial habenula and the raphe but is absent in cortex. The occurrence of the receptor in the midbrain raphe, where the cell bodies of 5-HT neurons are located raises the possibility that it may directly or indirectly influence the activity of 5-HT neurons, and thus the levels of 5-HT in the target structures. On the other hand, the 5-HT_5B_ receptor mRNA is present throughout the rat brain, with higher levels in the hippocampus, hypothalamus, pons, and cortex (Erlander et al., 1993).

The richest brain areas in 5-HT_6_ receptor mRNA are the ventral striatum and adjacent areas (nucleus accumbens, olfactory tubercle, islands of Calleja) as well as the dorsal striatum (caudate-putamen). High levels of 5-HT_6_ receptor mRNA are also found in the hypothalamus and the hippocampus, whereas the cerebral cortex, the substantia nigra, and the spinal cord contain low/moderate levels of the transcript (Gerard et al., 1996). Immunohistochemical studies have confirmed a similar distribution of the receptor protein, although the PFC shows a labeling density greater than that of the mRNA and similar to that of the hippocampus (Gerard et al., 1997).

Finally, the 5-HT_7_ receptor mRNA is localized to discrete regions of the rodent brain. Higher levels are present in the thalamus and the hippocampus whereas moderate levels are seen in the septum, the hypothalamus, the centromedial amygdala, and the periaqueductal gray. Autoradiographic studies indicate the presence of a similar distribution of the binding sites, in the cortex, septum, thalamus, hypothalamus, centromedial amygdala, periaqueductal gray, and superior colliculus (Gustafson et al., 1996). A similar distribution of 5-HT_7_ receptor was reported in human brain (Martin-Cora and Pazos, 2004). Interestingly, the 5-HT_7_ receptor is also localized in the raphe nuclei in both rodent and human brain, which has raised interest of targeting 5-HT receptors as a potential new mechanism to control brain's 5-HT levels by regulating the neuronal activity of the ascending 5-HT systems.

In summary, 5-HT released from axons innervating the cerebral cortex can modulate the activity of cortical neurons through several distinct receptors. However, with few exceptions (see above) little is known about the cellular phenotype of the neurons expressing 5-HT receptors, their precise distribution in cortical layers and the proportion of neurons of each type (e.g., pyramidal, stellate, or GABAergic neurons) expressing the receptor subtypes. The knowledge of these data is deemed important to identify the cellular elements and local circuitry involved in the cortical actions of 5-HT.

## Role of 5-HT receptors on cortical neuron activity

### 5-HT_1A_ receptors

The 5-HT_1A_ receptor has been characterized biochemically and electrophisiologically as being coupled to the Gi/o family of heterotrimeric G proteins. Gi/o proteins coupled to 5-HT_1A_ receptors are composed of pertussis toxin sensitive αi/αo subunits. This coupling mechanism was demonstrated *in vivo* and *in vitro* in the DR (Innis and Aghajanian, 1987). In hippocampal cells (as well as in 5-HT cells) a similar G protein couples 5-HT_1A_ and GABA_B_ receptors to potassium channels (Andrade et al., 1986).

5-HT_1A_ receptors are also coupled to potassium and—to a lesser extent—calcium channels. Intracellular current-clamp recordings in slices containing the DR had indicated that the 5-HT-mediated inhibition is mediated by an enhancement of the inward rectifying potassium conductance (Aghajanian and Lakoski, 1984; Williams et al., 1988). Exogenous application of 5-HT and 5-HT_1A_ agonists also elicit membrane potential hyperpolarization and decreased the membrane input resistance in DR 5-HT neurons *in vitro* (Aghajanian and Lakoski, 1984; Sprouse and Aghajanian, 1987), leading to an overall reduction in the probability of action potential firing. Similar effects to 5-HT_1A_ receptor activation have been reported in other neuronal types, such as hippocampal pyramidal cells (Andrade and Nicoll, 1987). 5-HT_1A_ receptors are also involved in the modulation of excitatory glutamatergic neurotransmission, since their activation suppresses AMPA-mediated signaling through the inhibition of CAMKII (Cai et al., 2002a) and reduces NMDA-mediated currents in PFC neurons (Zhong et al., 2008).

**Figure 3:**
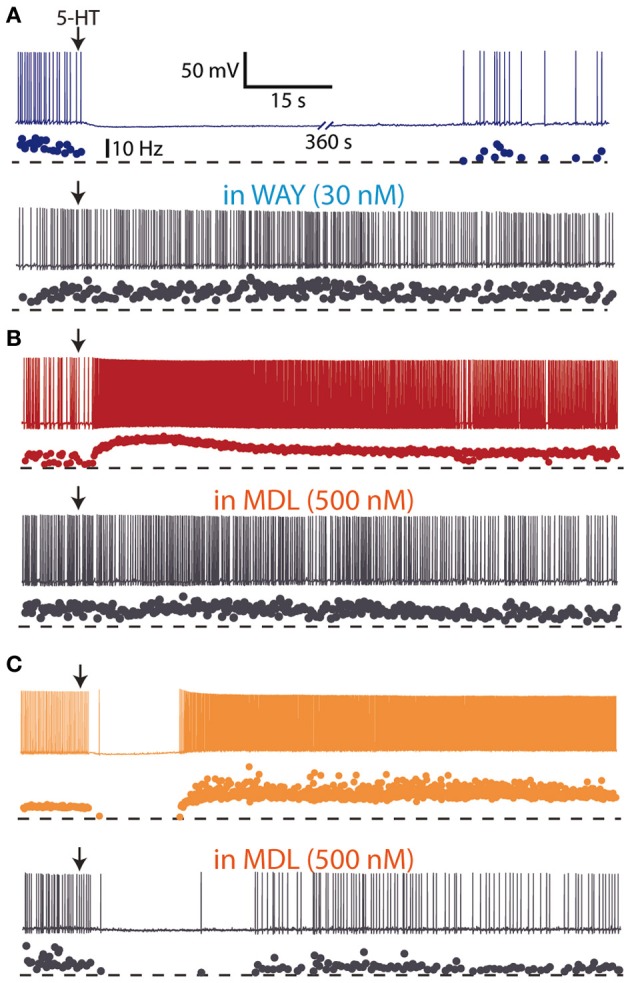
**Hyperpolarizing and depolarizing effects of 5-HT in layer V pyramidal neurons of medial prefrontal cortex slices from mice. (A)** Focal (local) application of 5-HT (100 μM) elicited a hyperpolarizing response which was blocked by the 5-HT_1A_ antagonist WAY 100635 (30 nM), suggesting the expression of 5-HT_1A_ receptors. **(B)** Focal application of 5-HT (100 μM) elicited a depolarizing response which was blocked by the 5-HT_2A_ antagonist MDL 11939 (500 nM), suggesting the expression of 5-HT_2A_ receptors. **(C)** Focal application of 5-HT (100 μM) elicited a biphasic response sensitive to the 5-HT_2A_ antagonist MDL 11939 (500 nM). Redrawn from Avesar and Gulledge (2012) with permission from A. Gulledge.

Early *in vivo* electrophysiological recordings showed that iontophoretic application of 5-HT excited and inhibited different cortical neurons (Krnjevic and Phillis, 1963; Roberts and Straughan, 1967), even though the major effect of 5-HT was inhibition of firing (Krnjevic and Phillis, 1963; Reader et al., 1979; Ashby et al., 1994; Zhang et al., 1994). Studies in the neocortex showed that 5-HT application also induced a hyperpolarization followed by a depolarization in a subpopulation of neurons (Davies et al., 1987; Tanaka and North, 1993). These effects were mediated, respectively by an action of 5-HT on 5-HT_1A_ and 5-HT_2_ receptors (Araneda and Andrade, 1991), and can be likely accounted for by the high co-expression of 5-HT_1A_ and 5-HT_2A_ receptors in cortical neurons (Amargós-Bosch et al., 2004). The three major actions of 5-HT on the activity of cortical neurons (inhibitions, excitations, and biphasic responses) have been recently found in layer V pyramidal neurons of mice PFC slices to be mediated by 5-HT_1A_ and 5-HT_2A_ receptors (Avesar and Gulledge, 2012) (Figure 3). Similarly, both hyperpolarizing (5-HT_1A_ receptor-mediated) and depolarizing (5-HT_2A_ receptor-mediated) responses to 5-HT have been demonstrated in human neocortex *in vitro* (Newberry et al., 1999).

In entorhinal cortex neurons recorded in the current clamp mode, 5-HT evoked a biphasic response consisting of a large amplitude hyperpolarization followed by a slowly developing, long lasting, and small amplitude depolarization. In voltage clamp, 5-HT consistently evoked an outward current by a smaller inward shift of holding current. The outward current was mediated by the activation of 5-HT_1A_ receptors (Ma et al., 2007).

Systemic administration of selective 5-HT_1A_ receptor agonists suppresses the firing activity of 5-HT neurons in the DR and MnR (Blier and de Montigny, 1987; Hajós et al., 1995; Casanovas et al., 2000) and hippocampal neurons (Tada et al., 1999). However, these agents appear to have a more complex effect on cortical (PFC) pyramidal neurons, with either a biphasic effect (increase in firing activity at lower doses followed by decrease at higher doses) or affecting different neuronal populations in a distinct manner (most neurons excited by 5-HT_1A_ agonists) (Borsini et al., 1995; Hajos et al., 1999; Diaz-Mataix et al., 2006; Lladó-Pelfort et al., 2010, 2012a,b). Thus, the systemic administration of the selective 5-HT_1A_ receptor agonist 8-OH-DPAT increased the firing activity of pyramidal neurons and reduced that of fast-spiking GABAergic interneurons, suggesting a preferential action of 5-HT_1A_ agonists on 5-HT_1A_ receptors in fast-spiking GABA interneurons, particularly at lower doses (Lladó-Pelfort et al., 2012b).

### 5-HT_1B/1D_ receptors

Anatomical and pharmacological evidence indicate that 5-HT_1B_ receptors have an axonal localization in different cerebral pathways and it exerts an inhibitory action on neurotransmitter release (Sari, 2004). Furthermore, electrophysiological evidence of the role of 5-HT_1B_ receptors in neuronal function is based on the assessment of inhibitory actions on evoked synaptic potentials or currents in target neurons whereas neurochemical studies have examined direct effects on neurotransmitter release.

**Figure 4:**
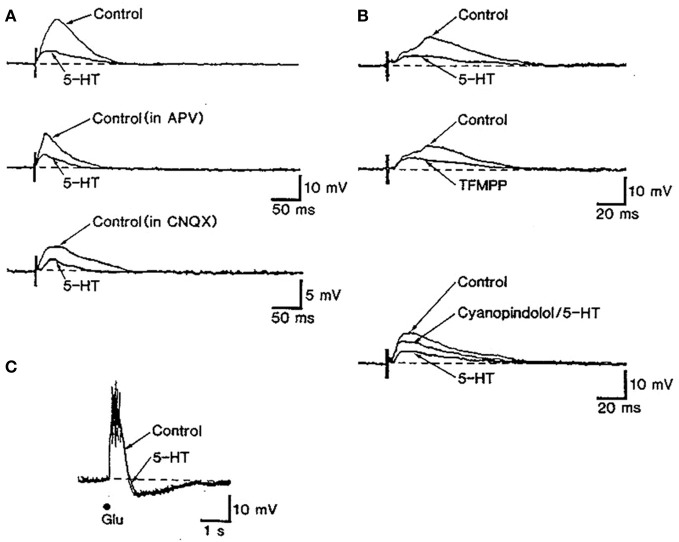
**The stimulation of 5-HT_1B_ receptors inhibits excitatory synaptic transmission in rat cingulate cortex. (A)** The stimulation of subcortical white matter elicited excitatory postsynaptic synaptic potentials (EPSP) dependent on NMDA and non-NMDA currents in in cingulate cortex pyramidal neurons. The action of 5-HT partially depressed EPSPs. **(B)** The reduction of EPSPs by 5-HT was mimicked by the non-selective 5-HT_1B_ agonist TFMPP and was antagonized by the 5-HT_1B_ antagonist cyanopindolol. **(C)** The depressing action of serotonin appears to be mediated by a presynaptic action of 5-HT since the depolarizing effect of glutamate was unaffected by 5-HT application. Reproduced with permission from Tanaka and North (1993).

The control of glutamate release by 5-HT_1B_ receptors has been described in different brain areas. In slices of cingulate cortex, 5-HT, acting on 5-HT_1B_ receptors, reduced the amplitude of NMDA and non-NMDA components of synaptic potentials recorded intracellularly in layer V pyramidal neurons (Tanaka and North, 1993) (Figure 4). It has been also reported that 5-HT_1B_ receptors mediate the 5-HT suppression of evoked fast excitatory postsynaptic current (evEPSC) in layer V pyramidal neurons in response to nearby electrical stimulation of cortical afferents (Lambe and Aghajanian, 2004).

### 5-HT_2A/2C_ receptors

5-HT_2A_ receptors are coupled to phospholipases through Gq proteins. Their activation entrains the production of IP3, diacylglycerol and the mobilization of intracellular Ca^2+^ stores. Furthermore, it is widely recognized that it is the main G-protein-coupled receptor through which 5-HT has excitatory actions. However, some aspects are highly controversial, including the localization of receptors responsible for the actions of 5-HT and 5-HT_2A_ agonists.

There is a substantial overlap between the localization of 5-HT axon terminals and 5-HT_2_ receptors in rat cortex (Blue et al., 1988). 5-HT_2A_ receptors are localized both to pyramidal neurons and GABAergic interneurons in the PFC (Willins et al., 1997; Jakab and Goldman-Rakic, 1998; Santana et al., 2004). 5-HT_2A_ receptors are highly expressed in large and medium-size parvalbumin- and calbindin-containing interneurons involved in the feed-forward inhibition of pyramidal neurons (Jakab and Goldman-Rakic, 2000; Puig et al., 2010). In the rat frontoparietal cortex, 5-HT axons are parallel to the apical dendrites of pyramidal neurons expressing 5-HT_2A_ receptors (Jansson et al., 2001). Additionally, a lower proportion of 5-HT_2A_ receptors was found presynaptically (Jakab and Goldman-Rakic, 1998; Miner et al., 2003).

**Figure 5:**
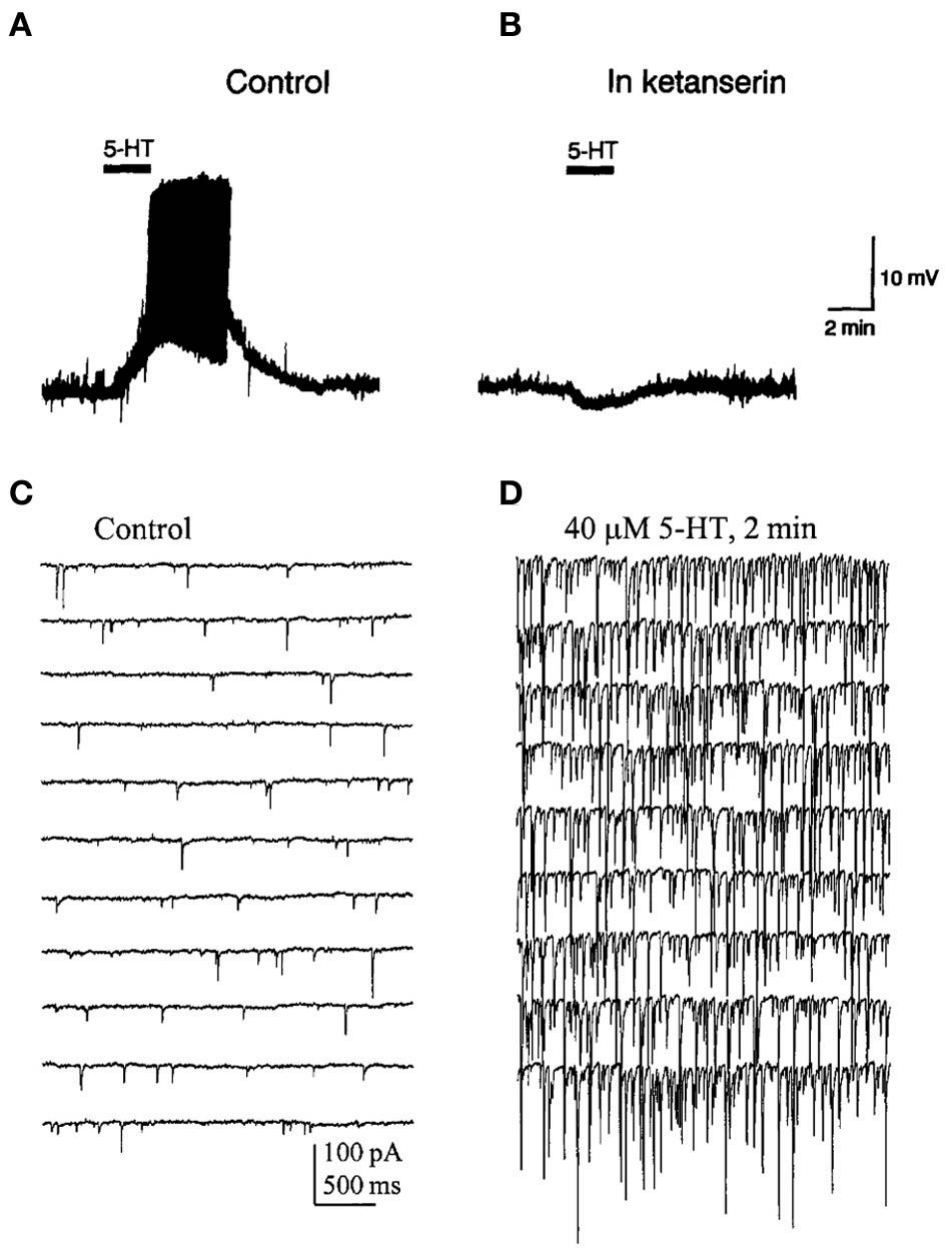
**(A)** Bath application of 5-HT evoked a depolarization and induced firing in a pyramidal neuron of the PFC. **(B)** Effect of ketanserin (5-HT_2A/2C_ receptor antagonist) on the 5-HT-induced membrane depolarization. Notice that in the presence of ketanserin 5-HT elicited a small hyperpolarization. **(C,D)** Spontaneous IPSCs recorded in a PFC pyramidal neuron in presence of AP-V and CNQX to block glutamatergic inputs. **(D)** IPSCs evoked by the bath application of 5-HT in the same recording conditions. Note the large increase in the frequency and amplitude of IPSCs induced by 5-HT. Reproduced with permission from Araneda and Andrade (1991) (panels **A,B**) and Zhou and Hablitz (1999) (panels **C,D**).

Activation of 5-HT_2A_ receptors exerts complex effects on the activity of PFC neurons. Thus, the microiontophoretic application of DOI suppressed the firing activity of putative pyramidal neurons in anesthetized rats but enhanced the excitatory effect of glutamate at low ejection currents (Ashby et al., 1990). *In vitro* recordings of identified pyramidal neurons in PFC slices have revealed that 5-HT_2A_ receptor activation increases spontaneous EPSCs and depolarizes the recorded cells (Araneda and Andrade, 1991; Tanaka and North, 1993; Aghajanian and Marek, 1997, 1999a,b; Zhou and Hablitz, 1999; Avesar and Gulledge, 2012) (Figures 3, 5). In addition, 5-HT can elicit 5-HT_2A_-mediated IPSCs through the activation of GABA synaptic inputs (Zhou and Hablitz, 1999), an effect that can be accounted for by the activation of 5-HT_2A_ receptors in GABAergic interneurons (Santana et al., 2004).

Importantly, recent studies have provided new insights into the role of 5-HT_2A_-mediated excitations and 5-HT_1A_-mediated inhibitions in PFC circuits. 5-HT generated 5-HT_2A_-mediated excitatory or biphasic responses in all callosal/commissural (COM) neurons responsive to 5-HT *in vitro*, whereas corticopontine-projecting neurons (CPn) were universally inhibited by 5-HT through 5-HT_1A_ receptors (Avesar and Gulledge, 2012). Additionally, cortico-mesencephalic pyramidal neurons respond to 5-HT_1A_ and/or 5-HT_2A_ receptor activation, as indicated by *in vivo* physiological (Puig et al., 2005) and pharmacological studies (Celada et al., 2001; Martín-Ruiz et al., 2001; Bortolozzi et al., 2005). Thus, 5-HT may exert different and projection-selective actions on PFC pyramidal neurons, activating cortico-cortical output channels, having mixed actions on some cortico-subcortical output channels and inhibiting other cortical pyramidal neurons, particularly CPn neurons. 5-HT_2A_ receptor-dependent serotonergic excitation of COM neurons may also be related to the parallel rostral-to-caudal gradients found for cortical 5-HT_2A_ receptors expression (Pazos et al., 1985; Weber and Andrade, 2010) and COM neuron density (Chao et al., 2009).

One of the most important mechanisms by which 5 HT increases pyramidal cell excitability seems to be mediated by the inhibition of the afterhyperpolarizating current (IAHP) typically observed after a burst of spikes in response to 5-HT_2_ receptor activation (Araneda and Andrade, 1991; Andrade, 1998). Accordingly, studies conducted in layer V neurons of PFC have identified 5-HT_2A_ receptors as the primarily receptor involved in the inhibitory effect of 5-HT on the slow afterhyperpolarizating current (IsAHP), suggesting also the contribution of additional 5-HT receptor subtypes (Villalobos et al., 2005). As AHP is involved in determining neuronal excitability, such an inhibition could contribute to regulate firing pattern activity of cortical neurons. Interestingly, the 5-HT_2A/2C_ receptor agonist DOI markedly affects the firing activity of PFC pyramidal neurons *in vivo* (Puig et al., 2003). Systemic DOI administration increased, decreased or left unaffected the activity of pyramidal neurons in PFC by a 5-HT_2A_ receptor-dependent mechanism. As observed for 5-HT (Zhou and Hablitz, 1999), inhibitory actions of DOI seems to be dependent on GABA_A_ receptor tone, suggesting the involvement of 5-HT_2A_ receptors in GABAergic interneurons.

There seems to be a tight link between 5-HT_2A_ receptors and glutamatergic transmission. Hence, the excitatory effects of DOI appear to involve interaction with glutamatergic transmission because DOI increases the excitatory effects of glutamate on prefrontal neurons (Ashby et al., 1989a, 1990). Likewise, the 5-HT_2A_ receptor-mediated EPSCs evoked by 5-HT in mPFC slices are occluded by blockade of AMPA receptors and mGluR II receptor activation (Aghajanian and Marek, 1997, 1999a,b). Moreover, the modulation of prefrontal NMDA transmission by 5-HT and DOB appears to involve pre- and postsynaptic 5-HT_2A_ receptors (Arvanov et al., 1999). Likewise, 5-HT modulates NMDA transmission in PFC via 5-HT_1A_ and 5-HT_2A_ receptors, with opposite actions of both receptors (Yuen et al., 2008; Zhong et al., 2008). Finally, the observation that the selective mGluRII agonist LY-379268 reversed the excitatory effect of DOI on pyramidal neurons *in vivo* is consistent with these *in vitro* observations (Puig et al., 2003).

It has been suggested that in mPFC 5-HT activates 5-HT_2A_ receptors located putatively on thalamocortical terminals to release glutamate and evoke EPSCs in pyramidal cells (Aghajanian and Marek, 1997). This interpretation was based on a number of observations, including the fact that this effect was antagonized by AMPA receptor antagonists and mGluR agonists (Aghajanian and Marek, 1997, 1999a,b). Similarly, the increase in pyramidal cell firing evoked by systemic DOI administration was dependent on glutamate inputs (Puig et al., 2003). However, the lesion of the thalamus (including the dorsomedial and centromedial nuclei which project to mPFC) (Berendse and Groenewegen, 1991; Fuster, 1997) did not abolish the excitatory effects of DOI on mPFC pyramidal neurons (Puig et al., 2003). Likewise, *in vitro* electrophysiological studies in 5-HT_2A_ receptor knockout mice are also discordant with the existence of presynaptic 5-HT_2A_ receptors in thalamocortical afferents (Béïque et al., 2007) and electron microscopy studies have failed to identify 5-HT_2A_ receptors in excitatory axonal terminals in the mPFC [most are located postsynaptically (Miner et al., 2003)]. Overall, these data have raised doubts about the presynaptic mechanisms responsible for the excitatory actions of 5-HT_2A_ receptors and suggest that postsynaptic receptors, which make up the majority of cortical 5-HT_2A_ receptors, are involved.

5-HT_2A_ receptors seem to exert a more marked depolarizing action in early stages of postnatal development, since the depolarizing action of 5-HT diminishes with age. Both 5-HT_2A_ and 5-HT_7_ receptors appears to underlie the depolarizing effect of 5-HT (see below) (Zhang, 2003; Béïque et al., 2004).

There is almost no evidence for a role of 5-HT_2C_ receptors in the modulation of cortical activity. 5-HT-induced slow excitations in prefrontal interneurons seem to be mediated exclusively by 5-HT_2A_ receptors, since the 5-HT_2C_ antagonist SB242084 failed to reduce 5-HT excitations previously blocked by a 5-HT_2A/2C_ antagonist (Puig et al., 2010). This agrees with the described primary expression of 5-HT_2C_ receptors in pyramidal neurons. Consistently, in the piriform cortex, it was reported that 5-HT could activate pyramidal neurons via 5-HT_2C_ receptors and GABAergic neurons via 5-HT_2A_ receptors (Sheldon and Aghajanian, 1991). However, the depolarizing action of 5-HT in layer V pyramidal neurons of the mPFC does not seem to depend on 5-HT_2C_ receptor activation since it was not blocked by the selective antagonist SB 242084 (Béïque et al., 2004). This inconsistency will require further investigation.

### 5-HT_3_ receptors

5-HT can mediate rapid excitatory responses through the activation of the 5-HT_3A_ receptor, a ligand-gated ion channel. These receptors may be involved in cortical actions of 5-HT, since some 5-HT_3_ receptor antagonists display procognitive effects (Staubli and Xu, 1995). These agents have been also reported to display anxiolytic and antipsychotic activity in animal models (Higgins and Kilpatrick, 1999) and to improve the therapeutic action of antipsychotic drugs in schizophrenic patients (Sirota et al., 2000). Likewise, the atypical antipsychotic clozapine is an antagonist of 5-HT_3_ receptors (Watling et al., 1989; Edwards et al., 1991).

Early microiontophoretic studies showed that 5-HT and 5-HT_3_ receptor agonists suppressed pyramidal neuron activity in rat PFC through the activation of 5-HT_3_ receptors by a direct action (Ashby et al., 1989b, 1991, 1992). However, more recent *in vitro* studies indicate that 5-HT may also increase pyramidal neurons IPSCs by activation of 5-HT_3_ receptors, likely as a result of a fast synaptic excitation of local GABAergic neurons (Zhou and Hablitz, 1999; Férézou et al., 2002; Xiang and Prince, 2003). The latter observations are consistent with the presence of 5-HT_3_ receptors in GABAergic interneurons in the rat telencephalon, including the PFC (Morales and Bloom, 1997; Puig et al., 2004). In macaque cortex, 5-HT_3_ receptors are expressed by a subpopulation of calbindin- and calretinin-positive interneurons (Jakab and Goldman-Rakic, 2000). *In vivo* studies also show the excitation of GABA interneurons in the mPFC through 5-HT_3_ receptors (Puig et al., 2004).

### Other 5-HT receptors

There is almost no information on the effects of 5-HT or selective agonists on cortical neurons through the activation of 5-HT_4_–5-HT_7_ receptors. For instance, it has been suggested that 5-HT_7_ receptor may play a role during early postnatal developing of cortical circuits. *In vitro* whole cell recordings revealed a shift in the effect of 5-HT on membrane potential across development according with coordinated changes in the expression and function of 5-HT_1A_, 5-HT_2A_, and 5-HT_7_ receptors. Hence, 5-HT in early postnatal days elicits a marked depolarization of pyramidal cells (dependent on 5-HT_2A_ and 5-HT_7_ receptors) which progressively shifts to hyperpolarization (mediated by 5-HT_1A_ receptors). This change appears to be mainly due to the loss of 5-HT_7_ receptors together with an increased function of 5-HT_1A_ receptors (Béïque et al., 2004).

The activation of 5-HT_4_ receptors has dual effects (enhancement or reduction) on GABA evoked currents in PFC pyramidal neurons, being the direction of the 5-HT_4_ receptor-mediated effect determined by neuronal activity. These observations suggest a flexible mechanism for 5-HT_4_ receptors to dynamically regulate synaptic transmission and neuronal excitability in the PFC network (Cai et al., 2002b; Yan, 2002). Also, extracellular recordings in rat frontal cortical slices have showed that bursting activity could be modulated by the application of the 5-HT_4_ agonist zacopride. Thus, perfusion of zacopride induces an increase on spontaneous bursting activity (Zahorodna et al., 2004).

Blockade of 5-HT_6_ receptors improves cortical performance in different learning and memory paradigms, an effect still poorly understood (Upton et al., 2008). Recent work using whole-cell path-clamp electrophysiological recordings showed that 5-HT_6_ agonists reversibly reduced spontaneous glutamatergic transmission in both striatal and layer V PFC pyramidal neurons, an effect prevented by preincubation in the selective 5-HT_6_ antagonists SB258585. Since no evidence for the expression of 5-HT_6_ receptors on glutamatergic neurons has been provided and it has been reported co-localization of this receptor on GAD immunoreactive neurons (Woolley et al., 2004) these data suggest that modulation of the glutamatergic transmission might be mediated by 5-HT_6_ receptors expressed by GABAergic neurons (Tassone et al., 2011).

### *In vivo* actions of endogenous serotonin on cortical neurons

**Figure 6:**
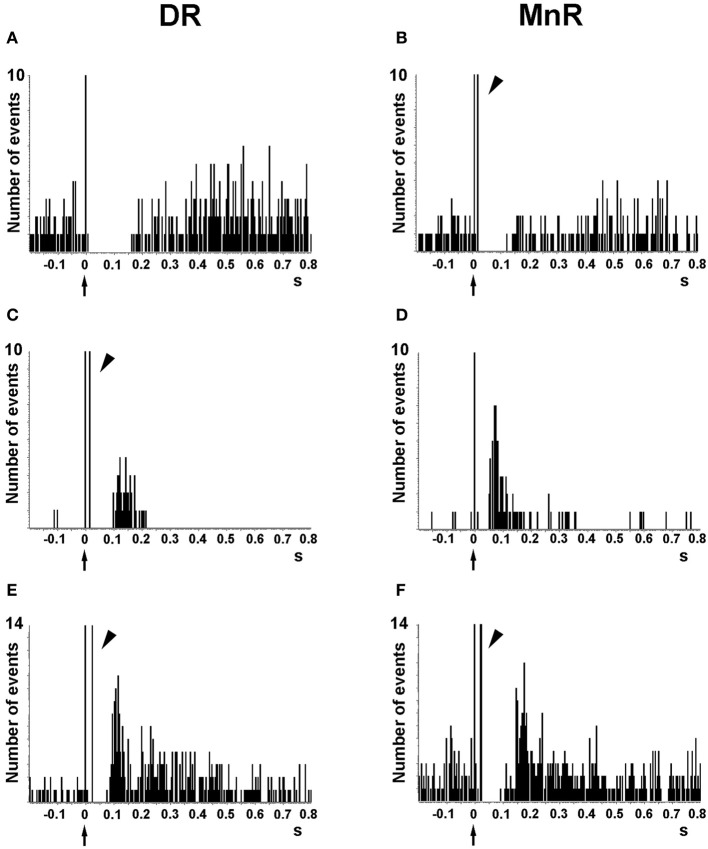
**Representative examples of the responses evoked in pyramidal neurons of the anterior cingulate and prelimbic areas of the mPFC by the electrical stimulation of the DR and MnR. (A,B)** Short latency long duration inhibitory responses. **(C,D)** Pure excitatory responses, which are often seen in pyramidal neurons with a low firing rate. These orthodromic excitations have a similar duration after DR or MnR stimulation but the latency is significantly lower after MnR stimulation, as in the example seen in the figure. **(E,F)** Examples of orthodromic excitations response preceded by short latency inhibitions (biphasic responses). Note the presence of antidromic spikes in the units recorded **(B,C,E,F)**, resulting from the presence of a dense connectivity between the PFC and the DR/MnR. The units in **(A)** and **(D)** were antidromically activated at currents higher than those used to evoke a pyramidal response. Each peristimulus time histogram consists of 110 triggers (2 min). Bin size = 4 ms. The arrow in the abcissa denotes the stimulus artifact (time 0). The arrowheads indicate antidromic spikes. Reproduced with permission from Puig et al. (2005).

Despite the wealth of *in vitro* studies on the actions of 5-HT on cortical neurons, the endogenous effects of 5-HT and its receptors in the regulation of cortical inhibitory and excitatory responses *in vivo* is not fully known. The effect of endogenous 5-HT on postsynaptic 5-HT_1A_ receptors has been typically examined in two forebrain areas, the CA region of the hippocampal formation and the mPFC using extracellular recordings. Electrical stimulation of the medial forebrain bundle inhibits hippocampal pyramidal neurons, an effect reversed by 5-HT_1A_ receptor blockade (Chaput and de Montigny, 1988). Similarly, as previously observed with the microiontophoretic application of 5-HT (see above), the electrical stimulation of DR/MnR at a physiological rate (~1 spike/s) mainly evoked inhibitory responses in PFC cells *in vivo*, which were partly or totally blocked by the selective 5-HT_1A_ antagonist WAY-100635 (Hajós et al., 2003; Amargós-Bosch et al., 2004; Puig et al., 2005, 2010). DR/MnR stimulation mainly evoked 5-HT_1A_-mediated inhibitory responses in two thirds of pyramidal neurons of the mPFC, identified by antidromic activation from the midbrain. The rest of responses were orthodromic excitations, either pure (13%) or preceded by short-latency inhibitions (20%, i.e., biphasic responses) (Puig et al., 2005) (Figure 6). Excitatory responses were blocked by the selective 5-HT_2A_ receptor antagonist M100907 (Amargós-Bosch et al., 2004; Puig et al., 2005).

Intriguingly, the proportion of excitatory responses was markedly lower than that of inhibitory responses despite the ~80% co-expression of 5-HT_1A_ and 5-HT_2A_ receptor mRNAs in PFC neurons (Amargós-Bosch et al., 2004). This observation agrees with the predominant inhibitory effects of 5-HT on cortical neurons observed *in vitro* (see above). The putative differential location of 5-HT_1A_ and 5-HT_2A_ receptors in different compartments of pyramidal neurons may perhaps account for the greater proportion of inhibitory responses. Should 5-HT_1A_ receptor be localized on axon hillocks, endogenous 5-HT would have a profound suppressing effect on action potential generation. Alternatively, a direct effect of 5-HT_1A_ receptor on ionic conductances (↑K^+^ currents) may favor inhibitory vs. excitatory actions of 5-HT mediated by 5-HT_2A_ receptors, which result from indirect and long lasting changes of similar ionic conductances into the opposite direction (↓ K^+^ currents, ↑ Ca2^+^ currents). Interestingly, 5-HT_1A_ and 5-HT_2A_ receptor mRNAs do not co-localize in parvalbumin-expressing inhibitory neurons of the PFC (Puig et al., 2010). This supports the notion that 5-HT_1A_ receptors in pyramidal neurons, by down-regulating action potential output, and 5-HT_2A_ receptors, by enhancing synaptic inputs onto the dendrites, exert a balanced modulation of cortical pyramidal networks across layers not available to small local interneurons (Puig and Gulledge, 2011).

In addition, inhibitory responses elicited in mPFC pyramidal neurons by raphe stimulation involve a GABAergic component since they were blocked by WAY-100635 but also by the GABA_A_ antagonist picrotoxinin (Puig et al., 2005). This GABAergic response may indeed result from the activation of local GABA interneurons by axon collaterals of pyramidal neurons projecting to midbrain. Likewise, 5-HT may also activate PFC GABAergic neurons through 5-HT_2A_ receptors thus inhibiting pyramidal cell activity, as observed *in vitro* (Zhou and Hablitz, 1999). The activation of 5-HT_3_ receptors is also likely, given their involvement in the excitatory responses of a subpopulation of GABAergic interneurons (Puig et al., 2004; see above). However, a third element seems also likely to explain the inhibition given the very short latency of inhibitory responses (9 ms on average) induced by DR/MnR stimulation. This latency is shorter than the time required for action potentials to travel along 5-HT axons from midbrain to PFC (orthodromic potentials; ~25 ms) or pyramidal axons from PFC to midbrain (antidromic potentials; ~15 ms). Overall, these observations would be consistent with the existence of a monosynaptic GABAergic projection from the midbrain raphe to mPFC, as suggested by anatomical studies (Jankowski and Sesack, 2002). This pathway would be analogous to the ascending GABAergic pathway between the ventral tegmental area and the mPFC or the nucleus accumbens—mesocortical and mesolimbic pathways, respectively—(Carr and Sesack, 2000), suggesting a common pattern of control of PFC by monoaminergic nuclei in which monoamine and projection GABA neurons would be involved.

Similarly to pyramidal neurons, endogenous 5-HT elicits 5-HT_1A_ receptor-mediated inhibitions and 5-HT_2A_ receptor-mediated excitations in PFC parvalbumin-expressing fast-spiking interneurons *in vivo* (Puig et al., 2010). There is also evidence that 5-HT_3_ receptors can activate GABA interneurons in the rat PFC *in vivo*. Physiological stimulation of the raphe nuclei excites local GABAergic neurons located in superficial layers (I–III) of the prelimbic and cingulate areas. These responses can clearly be distinguished from 5-HT_2A_ receptor-mediated excitations because they (a) have shorter onset latency and duration than 5-HT_2A_ receptor-mediated excitations, (b) show a higher concordance rate (number of spikes evoked/number of stimulus delivered in DR/MnR), and (c) are blocked by of the 5-HT_3_ receptor antagonists ondansetron and tropisetron (Puig et al., 2004; Puig and Gulledge, 2011).

In summary, three possibilities (differential localization and/or control of ion flows by 5 HT_1A_ and 5-HT_2A_ receptors in pyramidal neurons, existence of a raphe-mPFC GABAergic pathway, and inhibitory action in pyramidal cells mediated by activation of excitatory 5-HT receptors on GABAergic interneurons), not mutually exclusive, may account for the predominantly inhibitory responses elicited by raphe stimulation on pyramidal neurons of the mPFC. To the best of our knowledge, *in vivo* responses to other 5-HT receptors present in low or moderate abundances in cortex, such as 5-HT_2C_, 5-HT_4_, 5-HT_6_, or 5-HT_7_ have not been reported so far.

## Serotonin modulates cortical network activity

Neuronal populations, via their anatomical and functional interconnections, can display sophisticated discharge patterns that arise from the synchronization of their activity. That is, they can form neural networks whereby synchronous activity can become oscillatory. Oscillatory activities, ranging from 0.1 up to few hundred cycles per second, generate small electrical waves detectable outside the skull through electroencephalographic (EEG) recordings or intracerebrally through local field potential (LFP) recordings. Oscillations at several frequency bands have been recorded in the neocortex during natural sleep, anesthesia, and alertness, where their presence tightly correlates with a variety of behavioral tasks. During slow-wave sleep (SWS) and deep anesthesia, slow waves (~2 Hz) and delta waves (1–4 Hz) are prominent (Steriade et al., 1993; Mukovski et al., 2007; Celada et al., 2008; Puig et al., 2010), which are critical for memory consolidation and learning (Stickgold, 2005; Marshall et al., 2006; Landsness et al., 2009). During wakefulness, cortical alpha (10–14 Hz) and gamma (30–80 Hz) waves correlate with the modulation of attention, memory, and learning (Fries et al., 2001; Jensen et al., 2002; Ward, 2003; Buschman and Miller, 2007; Fries, 2009; Siegel et al., 2009; Benchenane et al., 2011; Bollimunta et al., 2011; Puig and Miller, 2012), whereas beta waves (15–30 Hz) also play a role in learning (Puig and Miller, 2012). Work reported over the last decade has suggested that the synchronization of neural activity in the neocortex and subcortical structures such as the hippocampus may indeed be critical for the normal processing of cognitive functions. In fact, schizophrenia patients, who show clear cognitive impairment (Elvevåq and Goldberg, 2000; Harvey et al., 2001), display abnormal cortical oscillatory activity in the slow and gamma frequency bands (Hoffmann et al., 2000; Spencer et al., 2003; Cho et al., 2006). Likewise, abnormal cortical oscillations can be observed in a variety of psychiatric disorders (see below). Considering the growing evidence pointing to an important role of cortical oscillations in cognition and the abundant psychiatric medication targeting the serotonergic system, the involvement of 5-HT in the generation and modulation of cortical oscillatory activities is of high interest, as it will help to identify new targets for psychiatric treatments.

### Serotonergic modulation of cortical oscillations

The seminal studies pioneered by Mircea Steriade and colleagues (Steriade et al., 1993; 1996, 2001; Steriade, 2006) described in detail the cellular mechanisms underlying the spontaneous slow rhythms (~2 Hz)—including slow and spindle waves—present in the neocortex during natural sleep. Slow waves reflect the spontaneous changes in membrane potential and synchronous firing of neuronal ensembles coordinated by an underlying slow oscillation. At a cellular level, they consist of an alternation between periods of activity (called UP states) and silence (DOWN states), that are not observed during wakefulness. UP and DOWN states reflect periods of membrane depolarization and hyperpolarization, respectively, within large neuronal networks (Steriade et al., 1993; Contreras and Steriade, 1995; Mukovski et al., 2007). Spontaneous low frequency oscillations (~1 Hz) have also been reported to occur in slices of the ferret visual cortex (Sanchez-Vives and McCormick, 2000), suggesting the existence of intrinsic mechanisms. To our knowledge, the role of 5-HT in the modulation of cortical slow oscillations during natural sleep has not been addressed, despite the marked activity changes of raphe 5-HT neurons during sleep and wakefulness.

**Figure 7:**
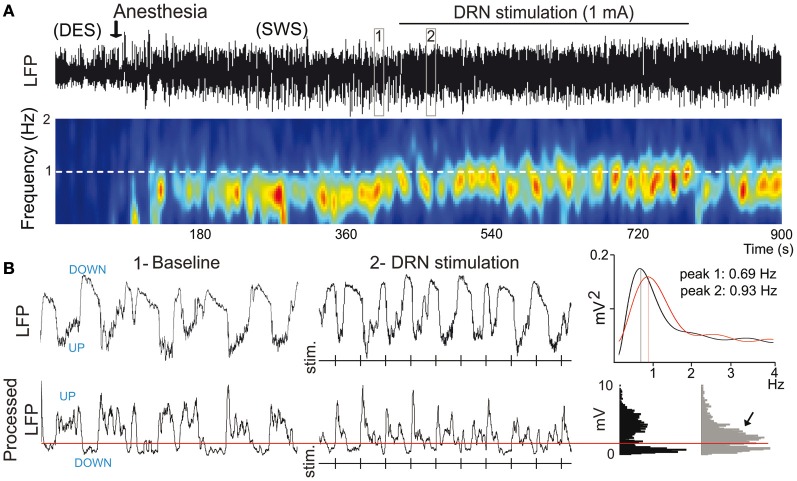
**5-HT modulates slow waves in the PFC. (A)** Stimulation of the dorsal raphe nucleus (DRN) increases the frequency of cortical slow waves (<1 Hz). Top, Local field potential (LFP) signal showing an epoch of desynchronization (absence of slow waves) preceding anesthesia-induced slow-wave sleep (SWS), during which the DRN was stimulated electrically at 1 Hz. Boxes 1 and 2 are expanded in **(B)**. Bottom, Change in power of slow waves over time (red indicates high power, blue low power). White dashed line marks the frequency of stimulation. Note that the predominant band (~0.7 Hz) increases in frequency toward the frequency of stimulation during DRN stimulation. **(B)** Top, expanded 10-s traces from **(A)**. Vertical lines correspond to times of DRN stimulation. Power spectra for 1 min segments that contain the 10-s traces in boxes 1 and 2 are shown on the far right. Bottom, LFPs were processed off-line for an accurate measure of UP-state duration. A threshold was set (red line) to discriminate UP states. Note the increase in UP-state potentials during the stimulations (arrow). Modified from Puig et al. (2010).

**Figure 8:**
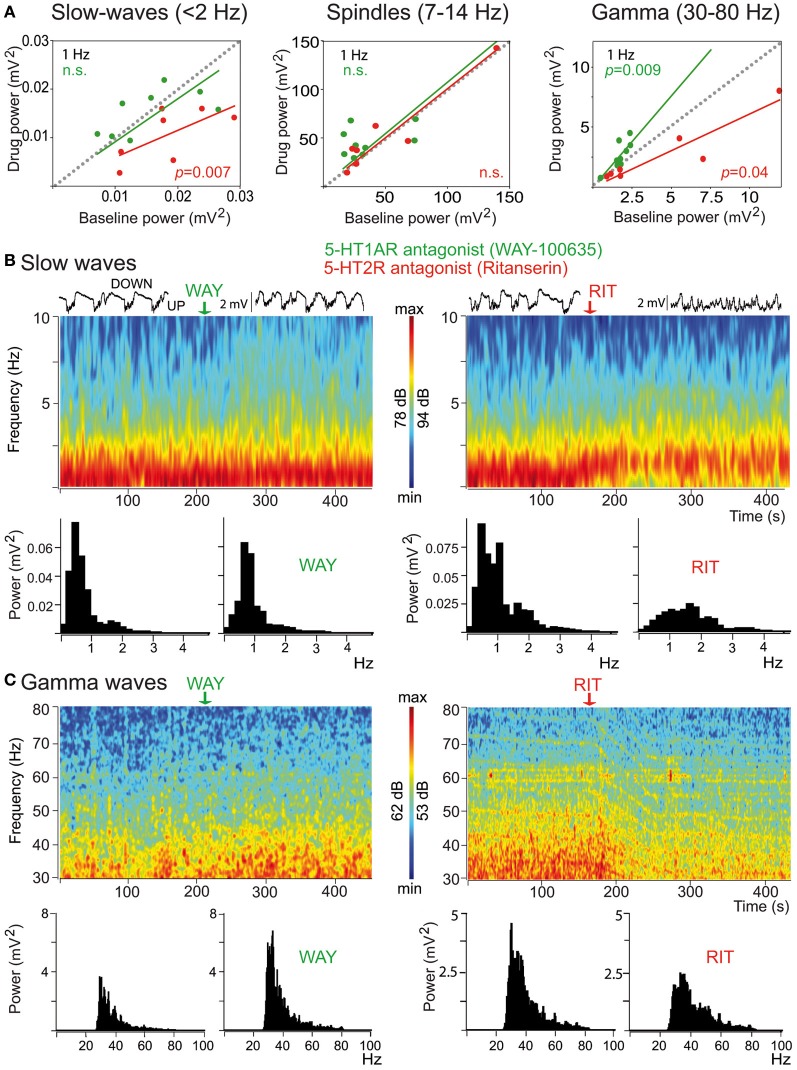
**Distinct modulation of cortical synchrony by 5-HT_1A_ and 5-HT_2A/2C_ receptors. (A)** Effects of the 5-HT_1A_ antagonist WAY (green) and the 5-HT_2A/2C_R antagonists ritanserin (RIT, red) on the baseline power (comparison of 30 s epochs before and after drug injection) of slow, spindle, and gamma oscillations. Note the opposing actions of the two drugs on the power of gamma oscillations. **(B)** Examples of the effects of WAY and RIT on the power of slow waves. Time-frequency plots and corresponding quantification of the power. WAY did not change significantly the power of slow-waves but RIT markedly reduced it. Red depicts high power, blue low power. Representative 10 s traces of the LFPs are shown on top of the spectrograms. **(C)** Examples of the effects of WAY and RIT on the power of gamma waves (same examples as in **B**). WAY augmented, whereas RIT reduced, the power of gamma oscillations. Modified from Puig et al. (2010).

Mild anesthetics, such as chloral hydrate, can reliably generate slow oscillations in the neocortex of laboratory animals that resemble the slow rhythms of natural SWS. This provides a more convenient preparation to examine how synchronous activity is modulated by 5-HT and its receptors. Endogenous 5-HT—released after electrical stimulation of the DR in the midbrain—modulates both the frequency and amplitude of cortical slow-like oscillations recorded in the PFC of anesthetized rats (Puig et al., 2010). 5-HT release produces a moderate increase in frequency by promoting rapid initiation of UP states, while reducing the amplitude and duration of DOWN states (Figure 7). This indicates that the activity of 5-HT neurons in the DR (and possibly MnR) may directly regulate the frequency of cortical slow oscillations by promoting UP states. Thus, despite most pyramidal neurons are inhibited by the physiological release of 5-HT (see above), 5-HT appears to have an excitatory effect on cortical networks *in vivo*, because UP states are generated by the synchronous depolarization of large ensembles of cortical neurons. In fact, a massive release of 5-HT in the cortex following high-frequency stimulation of the DR completely suppresses cortical slow waves by promoting a long-lasting depolarization and elimination of DOWN states (Puig et al., 2010). These excitatory influences of 5-HT on cortical slow oscillations may be accomplished via 5-HT_2A_ receptors (Celada et al., 2008; Puig et al., 2010). Hence, pharmacological stimulation of 5-HT_2A_ receptors with the hallucinogen and 5-HT_2A_ receptor agonist DOI and the blockade with the 5-HT_2*A*/2*C*_ antagonist ritanserin desynchronize slow waves in rat PFC (Figure 8), suggesting that a balanced stimulation of 5-HT_2A_ receptors is critical for a stable synchronization of cortical slow waves.

Much less is known about the role of 5-HT in the modulation of high-frequency oscillations, especially for the alpha (10–14 Hz) and beta (15–30 Hz) bands. A correlation between increases in the power of alpha oscillations in the ventral PFC and increased levels of 5-HT in whole blood has been found (Fumoto et al., 2010; Yu et al., 2011). Some more information has been reported on gamma oscillations. During natural sleep and anesthesia, gamma oscillations (30–100 Hz) are present in cortex along with slow waves (Steriade, 2006; Puig et al., 2010; Massi et al., 2012), although their specific involvement in cortical processing is poorly understood. Gamma oscillations are generated by the synchronous firing of fast-spiking interneuron networks (Cardin et al., 2009; Sohal et al., 2009), which exert potent inhibition onto pyramidal neurons and other fast-spiking neurons.

Interestingly, 5-HT exerts a strong modulation of gamma oscillations in the PFC of anesthetized rats via 5-HT_1A_ and 5-HT_2A_ receptors (Puig et al., 2010). Specifically, blockade of 5-HT_1A_ receptors increases the amplitude of cortical gamma waves, the discharge rate of 5-HT_1A_-expressing fast-spiking interneurons, and sharpens the synchronization of these neurons to gamma cycles. By contrast, blocking 5-H_2A_ receptors decreases cortical gamma oscillations and desynchronizes 5-HT_2A_-expressing fast-spiking interneurons from gamma waves. In other words, endogenous 5-HT can dampen or enhance gamma oscillations by reducing or increasing the activity and synchronization of 5-HT_1A_- and 5-HT_2A_-expressing fast-spiking interneurons, respectively. Overall, 5-HT's major effect is a reduction of cortical gamma oscillations during sleep-like epochs. Further investigations should establish whether these actions also occur during wakefulness.

### Relevance for psychiatric disorders

Abnormal oscillatory activities in the cortex have been observed in a number of neurological and psychiatric disorders (Basar and Güntekin, 2008). For example, the synchronization of slow (<1 Hz), delta (1–4 Hz) and gamma (30–80 Hz) oscillations is reduced in schizophrenia, major depression, and bipolar disorder (Keshavan et al., 1998; Hoffmann et al., 2000; Spencer et al., 2003; Cho et al., 2006; Uhlhaas and Singer, 2006). Impaired gamma oscillations and synchrony have also been reported in schizophrenia patients as well, suggesting the existence of network alterations (Spencer et al., 2003; Cho et al., 2006; Uhlhaas and Singer, 2006; Basar and Güntekin, 2008; Gonzalez-Burgos and Lewis, 2008; Gonzalez-Burgos et al., 2010).

Interestingly, patients with depression that do not respond to selective serotonin reuptake inhibitors (SSRIs) have alterations in alpha power in some cortical areas compared with responders and healthy control subjects (Bruder et al., 2008), suggesting some relationship between 5-HT levels and the amplitude of alpha oscillations.

Animal models of psychiatric disorders are starting to shed new light into the contribution of 5-HT to normal and abnormal cortical oscillations. Importantly, they are providing valuable information to better understand the cellular mechanisms by which psychiatric medication compensates for imbalances in network activity. For instance, the hallucinogen and preferential 5-HT_2A_ receptor agonist DOI disrupts low-frequency oscillations in the PFC of anesthetized rats (Celada et al., 2008), an effect reversed by antipsychotic drugs with different pharmacological targets. While the effect of clozapine can be interpreted by its competition with DOI at 5-HT_2A_ receptors, the effect of haloperidol must necessarily be interpreted at the network level, given its inability to occupy 5-HT_2A_ receptors at the dose used. A reduction in slow wave activity has been detected in patients with schizophrenia during sleep (Hoffmann et al., 2000); hence, a potential source of this decrease could be an unbalanced stimulation of cortical 5-HT_2A_ receptors.

Interestingly, the disruption of PFC activity evoked by DOI is similar—yet of smaller magnitude—to that produced by the NMDA receptor antagonist phencyclidine (PCP) (Kargieman et al., 2007). Interestingly, PCP effect is also reversed by haloperidol and clozapine, which suggests a link between the disrupting action of PCP and DOI on PFC activity and their psychotomimetic activity. Likewise, the reversal of this effect by two antipsychotic drugs with different primary targets strongly suggests a relationship with their therapeutic action. Despite its low *in vitro* affinity for 5-HT_1A_ receptors, the reversal by clozapine of PCP effects depends on the *in vivo* stimulation of such receptors (Kargieman et al., 2012), as previously observed for the antipsychotic-evoked release of dopamine in mPFC (Diaz-Mataix et al., 2005).

Collectively, these studies highlight the importance of 5-HT in regulating cortical network activity, as well as the complexities of the alterations present in psychiatric disorders and the compensatory mechanisms mediating the action of psychiatric medication. Detailed knowledge of the cellular and circuit mechanisms underlying serotonergic modulation of cortical oscillations in health and disease could provide valuable information for our understanding of why many schizophrenia and other psychiatric treatments are largely ineffective at restoring PFC function.

## Conclusions

The assessment of the *in vivo* and *in vitro* actions of 5-HT on cortical neurons and networks has revealed a complex pattern of action. 5-HT can hyperpolarize pyramidal neurons through the activation of 5-HT_1A_ receptors, an action that results from the opening of G protein-coupled inward rectifying K^+^ channels. This effect is followed by a reduction of the firing activity of pyramidal neurons. At the same time, 5-HT can depolarize the same neurons through 5-HT_2A_ receptors and increase their excitability. These two receptors appear to be the main players for the postsynaptic actions of 5-HT in the cerebral cortex. *In situ* hybridization studies have revealed the concurrent presence of 5-HT_1A_ and 5-HT_2A_ receptor mRNAs in a large proportion (~80%) of neurons in the PFC. Although several hypotheses have been put forward, it is yet unclear what determines whether a given pyramidal neuron responds to 5-HT with an excitation or an inhibition, although the latter responses predominate, both *in vitro* and *in vivo*. However, the fact that 5-HT induces depolarizing actions on slow oscillations *in vivo* suggests that it can exert excitatory effects on cortical neural networks independent from action potential generation, a view awaiting further confirmation. The role of these two receptors in the modulation of the activity of GABAergic interneurons is still poorly understood. A significant proportion of these neurons, including fast-spiking interneurons, located in layers II–VI express 5-HT_1A_ and/or 5-HT_2A_ receptors; yet so far, there are no studies examining the role of both receptors in the modulation of ion currents. *In vivo*, 5-H_1A_ receptors decrease, whereas 5-HT_2A_ receptors increase, spiking rate of fast-spiking interneurons in the PFC (Puig et al., 2010).

On the contrary, there is a reasonable knowledge on the role of 5-HT_3_ receptors in the control of the activity of GABAergic interneurons. It seems like there is a segregation of 5-HT_1A/5-HT_2A__ receptors on one side, expressed likely in parvalbumin- and calbindin-containing interneurons and 5-HT_3_ receptors, expressed in calretinin- and (to a lesser extent) calbindin-containing neurons. Moreover, interneurons expressing 5-HT_3_ receptors are localized mostly in layers I-III, which suggests a role in the modulation of inputs reaching the tufts and upper segments of the apical dendrites of pyramidal neurons.

**Figure 9:**
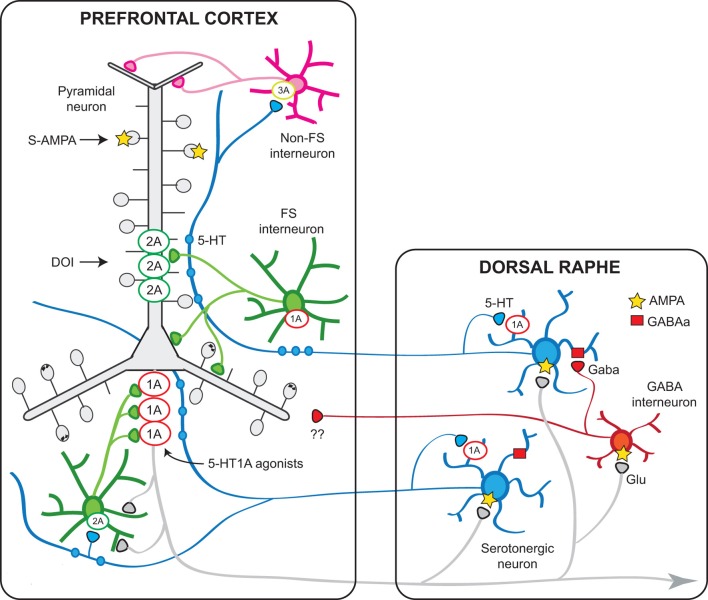
**Schematic representation of the relationships between the mPFC and the DR involving 5-HT_1A_ and 5-HT_2A_ receptors.** Pyramidal neurons in the mPFC project densely to the DR/MnR and modulate the activity of serotonergic neurons via direct and indirect influences (Celada et al., 2001). In turn, endogenous 5-HT modulates pyramidal cell activity through the activation of various receptors expressed in the neocortex, of which 5-HT_1A_ and 5-HT_2A_ receptors play a major role. The latter receptors are particularly enriched in apical dendrites of pyramidal neurons where they can facilitate AMPA inputs. A smaller population of 5-HT_2A_ receptors are expressed by GABA interneurons, including fast-spiking (FS) interneurons. Pyramidal 5-HT_1A_ receptors may be localized in the axon hillock, together with GABAA receptors activated by chandelier axons (Azmitia et al., 1996; De Felipe et al., 2001; Cruz et al., 2004) or in the somatodendritic compartment (Riad et al., 2000). It is possible that 5-HT axons reaching the cortex at different levels may exert distinct effects on pyramidal neurons, depending on a precise topology between certain 5-HT neurons or neuronal clusters within the DR/MnR and 5-HT_1A_- or 5-HT_2A_-receptor-rich compartments, in agreement with anatomical studies showing an association between 5-HT axons and such receptor-rich areas (De Felipe et al., 2001; Jansson et al., 2001). Also, 5-HT axons reaching upper layers, including layer I, may activate 5-HT_3_ receptors located on GABAergic non-FS interneurons to modulate inputs onto the tufts and most distant segments of apical dendrites of pyramidal neurons. 5-HT_1B_ receptors are present on serotonergic axons (not shown) and in axons of other neuronal types (e.g., glutamatergic) where they regulate neurotransmitter release and modulate synaptic activity. The scheme shows also the putative GABAergic projections from DR/MnR to the mPFC suggested by electrophysiological and anatomical studies (see text).

Another 5-HT receptor for which a role (yet still poorly characterized) has been attributed is the 5-HT_1B_ receptor, whose activation by 5-HT can presynaptically modulate GABAergic and glutamatergic inputs onto pyramidal neurons. Figure 9 shows a schematic representation of the PFC—raphe circuit with the most important receptors involved in the serotonergic actions in PFC and their presumed localization.

Unfortunately, there is a poor knowledge of the actions of 5-HT on other receptors, some of which are expressed in significant amounts in the neocortex. 5-HT_2C_ receptors can modulate the activity of pyramidal neurons in piriform cortex, but this does not seem to be the rule in neocortex. On the other hand, the neuronal depolarization induced by 5-HT_7_ receptor activation disappears few weeks after birth, which suggests a role in development but not in adulthood. Further studies are indeed required to clarify the complex role of 5-HT in the modulation of cortical activity. Current and new knowledge in this area will help to understand the involvement of 5-HT in cortical functions, notably those in PFC, a brain region highly enriched of 5-HT elements and involved in critical brain functions such as cognition and emotional control, among others.

### Conflict of interest statement

The authors declare that the research was conducted in the absence of any commercial or financial relationships that could be construed as a potential conflict of interest.
